# Urinary Obstruction Secondary to Fecal Impaction: An Unusual Presentation of Stercoral Colitis

**DOI:** 10.7759/cureus.108795

**Published:** 2026-05-13

**Authors:** Neeraj Joshi, Bennet Jose Kandathy, Sukriti Bansal, Oluwafemi Akanbi, Shihab Bappy

**Affiliations:** 1 Cardiology/Internal Medicine, Queen Elizabeth The Queen Mother (QEQM) Hospital, Margate, GBR; 2 Medicine, Queen Elizabeth The Queen Mother (QEQM) Hospital, Margate, GBR; 3 General Medicine, Queen Elizabeth The Queen Mother (QEQM) Hospital, Margate, GBR

**Keywords:** antipsychotic medication, colitis, constipation misperception, drug-related side effects and adverse reactions, fecaloma, obstructive hydronephrosis, stercoral colitis

## Abstract

Stercoral colitis is an inflammatory complication of prolonged fecal impaction, with presentations ranging from mild colonic inflammation to life-threatening perforation. Although gastrointestinal manifestations are well recognized, secondary involvement of adjacent pelvic structures due to mass effect remains underreported and may contribute to atypical clinical presentations. An elderly woman with paranoid schizophrenia receiving long-term antipsychotic therapy presented with a three-day history of lower abdominal pain, distension, and urinary retention. Although there was no documented history of recurrent constipation, bowel obstruction, or prior related hospital admissions, chronic subclinical constipation could not be excluded, given the background of prolonged antipsychotic use. Cross-sectional CT of the abdomen and pelvis demonstrated significant fecal impaction within the sigmoid colon and rectum, associated with bowel wall thickening and pericolonic fat stranding consistent with stercoral colitis. The fecaloma exerted marked extrinsic compression on the urinary bladder, resulting in bilateral hydronephrosis and hydroureter in the absence of intrinsic urological pathology, consistent with obstructive uropathy secondary to fecal impaction. This case highlights an uncommon but clinically important mechanism by which fecal impaction complicated by stercoral colitis may result in urinary tract obstruction. It emphasizes the importance of considering extrinsic compressive etiologies in the evaluation of urinary retention, particularly in patients with neuropsychiatric comorbidities and exposure to medications associated with impaired bowel motility. Early recognition and timely bowel decompression are critical for clinical resolution and may reduce the need for invasive urological intervention.

## Introduction

Stercoral colitis is an uncommon, potentially life-threatening inflammatory colitis caused by prolonged fecal impaction and increased intraluminal pressure leading to ischemic injury of the colonic wall [1]. Although increasingly recognized with the widespread use of cross-sectional imaging, its true prevalence remains uncertain, largely due to underdiagnosis and overlap with more common causes of acute abdomen. A recent retrospective review from 21 emergency departments in the United States reported stercoral colitis in approximately 0.003% of all CT of the abdomen/pelvis studies and 0.008% of scans performed in patients aged more than 65 years [2]. Despite its relative rarity, stercoral colitis carries significant clinical importance because of its potential for rapid progression and high mortality, particularly when complicated by perforation, where reported mortality rates may reach 32% to 60% [3].

The pathophysiology of stercoral colitis is driven by persistent fecal stasis, most often within the rectosigmoid colon, where desiccated stool forms a fecaloma. Progressive accumulation leads to rising intraluminal pressure, impairing mucosal perfusion and resulting in localized ischemia and inflammation. If unrelieved, this process may progress to ulceration, transmural necrosis, and, ultimately, perforation [4]. This sequence, from constipation to fecal impaction, followed by stercoral colitis, highlights the importance of recognizing it not as a primary disease, but as a downstream complication of disordered bowel motility.

Multiple predisposing factors contribute to this process. Medications are particularly relevant, especially those with anticholinergic or central sedative effects that reduce gastrointestinal motility. Antipsychotic agents such as olanzapine, along with anticholinergics such as procyclidine, are well-recognized contributors to severe constipation and fecal impaction [5,6]. In addition, systemic factors such as dehydration, reduced mobility, and low-fiber intake further exacerbate stool stasis. Neuropsychiatric and neurological conditions, including schizophrenia, dementia, and autonomic neuropathies, compound this risk by impairing bowel awareness, altering pain perception, and delaying presentation [4,7]. This interplay is particularly relevant in our patient, whose underlying psychiatric condition and long-term medication use created a permissive environment for severe fecal loading.

Clinically, stercoral colitis spans a wide spectrum, from mild inflammatory changes to catastrophic complications. Stercoral perforation, the most feared outcome, accounts for approximately 3% of all colonic perforations and is associated with mortality rates as high as 30-60% [8,9]. Beyond perforation, complications such as sepsis, ischemic colitis, and acute kidney injury are well described. However, the potential for clinically significant mass effect from a large fecaloma is less frequently recognized. Within the confined pelvic cavity, the rectosigmoid colon lies immediately posterior to the urinary bladder [10], while the distal ureters course along the lateral pelvic walls before entering the posterolateral aspect of the bladder [11]. Mass-forming pathology in this region via pelvic mass effect [12] has been reported to cause hydronephrosis through extrinsic ureteric compression. By analogy, a markedly distended rectosigmoid segment containing a large fecaloma can produce a similar mass effect on the bladder and distal ureters, producing extrinsic compression and functional urinary outflow obstruction in the absence of intrinsic urological pathology. This mechanism remains sparsely reported and is often overlooked in the differential diagnosis of obstructive uropathy [3].

CT of the abdomen and pelvis remains the diagnostic modality of choice. It allows reliable differentiation between uncomplicated fecal impaction and stercoral colitis, while simultaneously identifying complications. Typical findings include colonic dilatation with fecaloma, bowel wall thickening, and surrounding fat stranding, with or without features of ischemia or perforation [4]. Given the frequently non-specific clinical presentation, early imaging is critical to establishing the diagnosis and guiding timely management.

Management of stercoral colitis is largely determined by disease severity. In uncomplicated cases, treatment is primarily conservative, focusing on relieving fecal impaction through laxatives, enemas, and manual or endoscopic disimpaction, alongside adequate hydration and monitoring. Antibiotics are reserved for patients with suspected systemic inflammatory response. Surgical intervention is indicated in the presence of complications such as perforation or peritonitis, or when conservative measures fail [9]. Outcomes are closely linked to the timing of recognition, with delayed diagnosis associated with significantly increased morbidity and mortality.

Against this background, we present the case of a patient with paranoid schizophrenia who developed acute urinary obstruction secondary to fecal impaction complicated by stercoral colitis. Cross-sectional imaging demonstrated significant fecal loading within the sigmoid colon and rectum, with associated inflammatory changes, accompanied by bilateral hydroureter and hydronephrosis in the absence of intrinsic urological pathology. This case highlights a likely underrecognized mechanism of urinary tract obstruction and underscores the importance of considering gastrointestinal pathology in atypical presentations of urinary retention, particularly in patients at high risk of severe constipation.

## Case presentation

A 76-year-old woman with a background of paranoid schizophrenia and prior fragility fractures, living independently, presented to the emergency department with a three-day history of progressive lower abdominal pain, increasing distension, vomiting, and acute urinary retention. There was no history of fever, dysuria, hematuria, recent urinary tract infection, trauma, or spinal symptoms to suggest infective or neurogenic causes. A review of prior medical records revealed no previous admissions for constipation, bowel obstruction, or similar abdominal presentations over the preceding seven to eight years.

Her regular medications included olanzapine 10 mg once daily (since 2017), procyclidine 5 mg three times daily (since 2022), atorvastatin 20 mg nightly, and alendronic acid 70 mg weekly. She denied chronic constipation, altered bowel habit, unintentional weight loss, anorexia, or rectal bleeding, and her psychiatric condition was reported as stable with no recent changes in therapy or functional status.

**Figure 1:**
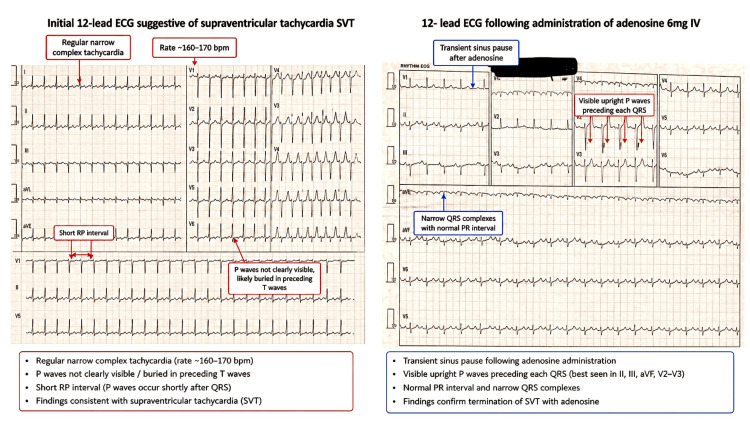
Electrocardiographic findings demonstrating supraventricular tachycardia and post-adenosine reversion to sinus rhythm. Initial 12-lead electrocardiogram demonstrating a regular narrow-complex tachycardia with a heart rate of approximately 160-170 beats per minute, short RP interval, and poorly visible P waves, consistent with supraventricular tachycardia. Repeat electrocardiogram following administration of 6 mg intravenous adenosine demonstrates a transient sinus pause with restoration of sinus rhythm, visible upright P waves preceding each QRS complex, and a normal PR interval with narrow QRS complexes.

On arrival, she appeared clinically dehydrated. Abdominal examination demonstrated generalized tenderness with visible distension, but no guarding or rebound to suggest peritonism, and bowel sounds were present. There was no renal angle tenderness, and cardiovascular, respiratory, and neurological examinations were otherwise unremarkable. Initial observations showed borderline hypotension (blood pressure: 101/49 mmHg) and marked tachycardia, with heart rates between 160 and 170 beats per minute. A 12‑lead electrocardiogram confirmed narrow‑complex supraventricular tachycardia, which reverted to sinus rhythm following administration of 6 mg intravenous adenosine (Figure 1).

Given the acute presentation, the initial differential diagnosis for an acute abdomen included diverticular disease, intra‑abdominal malignancy, bowel obstruction, and obstructive uropathy. Infective causes such as pyelonephritis were considered less likely in the absence of fever, lower urinary tract symptoms, or renal angle tenderness. Medication‑related constipation was initially thought to be less likely given the absence of prior episodes despite long‑term antipsychotic therapy. The abrupt onset of symptoms and lack of red flag features, including weight loss or rectal bleeding, reduced the clinical suspicion of an underlying malignancy.

Baseline investigations comprised full blood count, inflammatory markers, renal function, thyroid function, electrolytes, venous blood gas, amylase, cardiac enzymes, chest radiography, and electrocardiography. These demonstrated an inflammatory response, with elevated C‑reactive protein of 93 mg/L, leukocytosis with a white blood cell count of 14.9 × 10⁹/L, and neutrophilia of 12.9 × 10⁹/L. Hemoglobin was mildly reduced at 111 g/L. Renal function was preserved (creatinine: 63 µmol/L, estimated glomerular filtration rate: 82 mL/minute/1.73 m²), and serum electrolytes (sodium: 133 mmol/L, potassium: 4.0 mmol/L) were within normal limits. Liver function tests were largely unremarkable apart from a mildly elevated alkaline phosphatase of 146 U/L and hypoalbuminaemia of 23 g/L, which was considered multifactorial and likely related to the acute inflammatory response and underlying frailty, with albumin acting as a negative acute‑phase reactant. There were no clinical features to suggest chronic liver disease, malabsorption, or occult malignancy during admission. Thyroid function (thyroid-stimulating hormone: 2.4 mIU/L, free thyroxine: 15 pmol/L) and cardiac biomarkers (troponin: 3 ng/L) were within the reference ranges (Table 1).

**Table 1:** Initial laboratory investigations on admission. Summary of initial laboratory investigations at presentation demonstrating an inflammatory response with elevated C-reactive protein and leukocytosis. Renal function and electrolytes were within normal limits despite clinical evidence of urinary obstruction. Thyroid function and cardiac biomarkers were unremarkable. WBC: white blood cell count; eGFR: estimated glomerular filtration rate; ALT: alanine aminotransferase; ALP: alkaline phosphatase; CRP: C-reactive protein; TSH: thyroid-stimulating hormone; free T4: free thyroxine

Parameter	Value	Units	Reference range
Creatinine	63	µmol/L	64–104 µmol/L
eGFR	82	mL/minute/1.73 m²	>60 mL/minute/1.73 m²
Sodium	133	mmol/L	133–146 mmol/L
Potassium	4.0	mmol/L	3.5–5.3 mmol/L
Phosphate	0.9	mmol/L	0.8–1.5 mmol/L
Magnesium	0.92	mmol/L	0.7–1.0 mmol/L
Albumin-adjusted calcium	2.4	mmol/L	2.2–2.6 mmol/L
Bilirubin	13	µmol/L	0–29 µmol/L
ALT	32	U/L	0–70 U/L
ALP	146	U/L	30–130 U/L
Albumin	23	g/L	35–50 g/L
CRP	93	mg/L	0–10 mg/L
WBC	14.9	×10⁹/L	4.0–11.0 × 10⁹/L
Neutrophils	12.9	×10⁹/L	2.0–7.5 × 10⁹/L
TSH	2.4	mIU/L	0.4–5 mIU/L
Free T4	15	pmol/L	9–19 pmol/L
Amylase	32	U/L	0–125 U/L
Creatine kinase	93	U/L	25–200 U/L
Troponin	3	ng/L	0–16 ng/L

**Figure 2:**
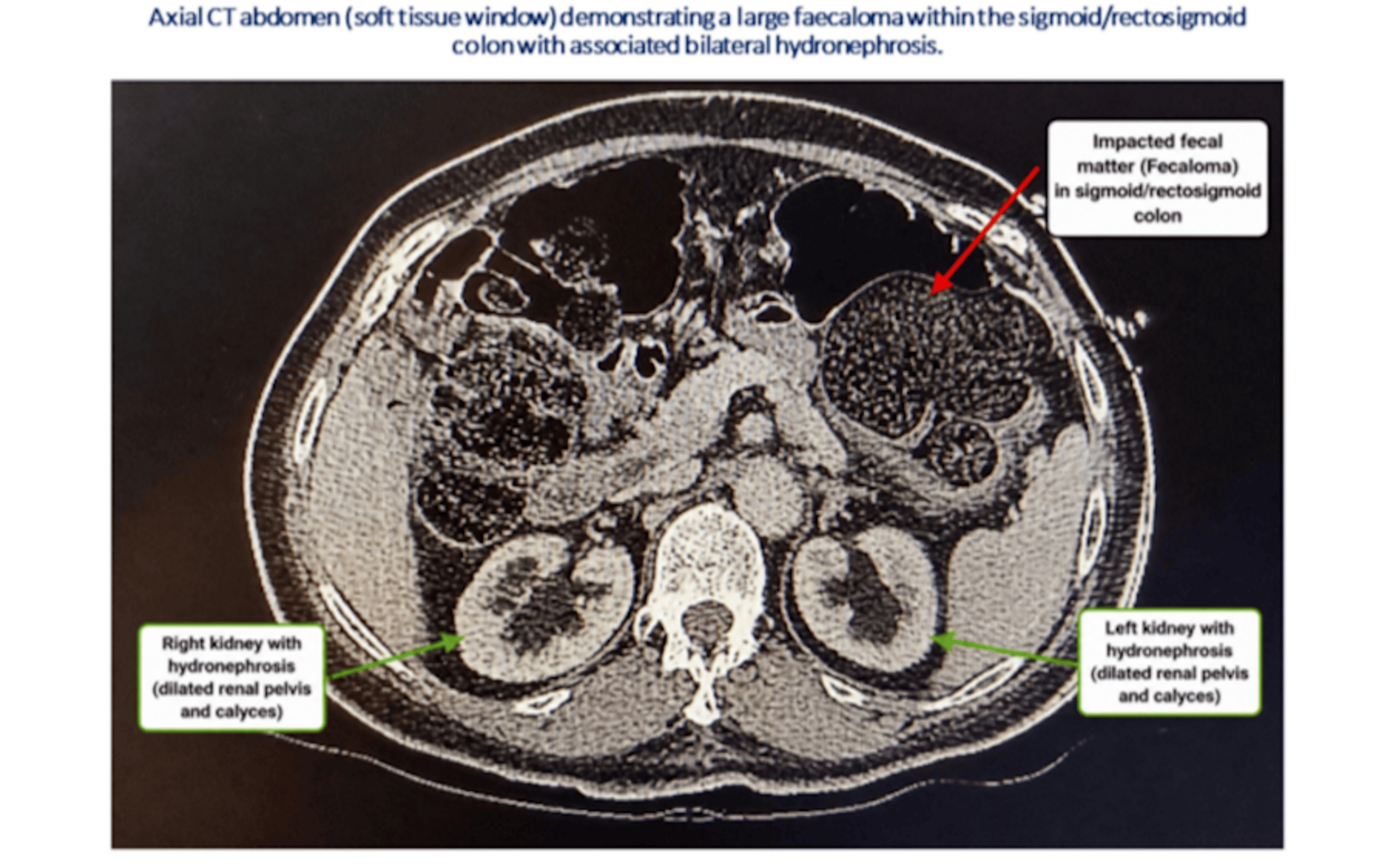
Contrast-enhanced CT of the abdomen (axial, soft-tissue window). Axial contrast-enhanced CT of the abdomen (soft-tissue window) demonstrating marked fecal loading with a large fecaloma within the sigmoid/rectosigmoid colon measuring approximately 7.1 × 6.2 cm, consistent with fecal impaction (red arrow). Associated bilateral hydronephrosis is present, evidenced by dilatation of the renal pelvis and calyces (green arrows) with renal pelvic dilatation measuring approximately 2.0 cm on the right and 1.9 cm on the left. Findings are in keeping with stercoral colitis causing extrinsic compression of the urinary bladder and distal ureters and resultant obstructive uropathy.

**Figure 3:**
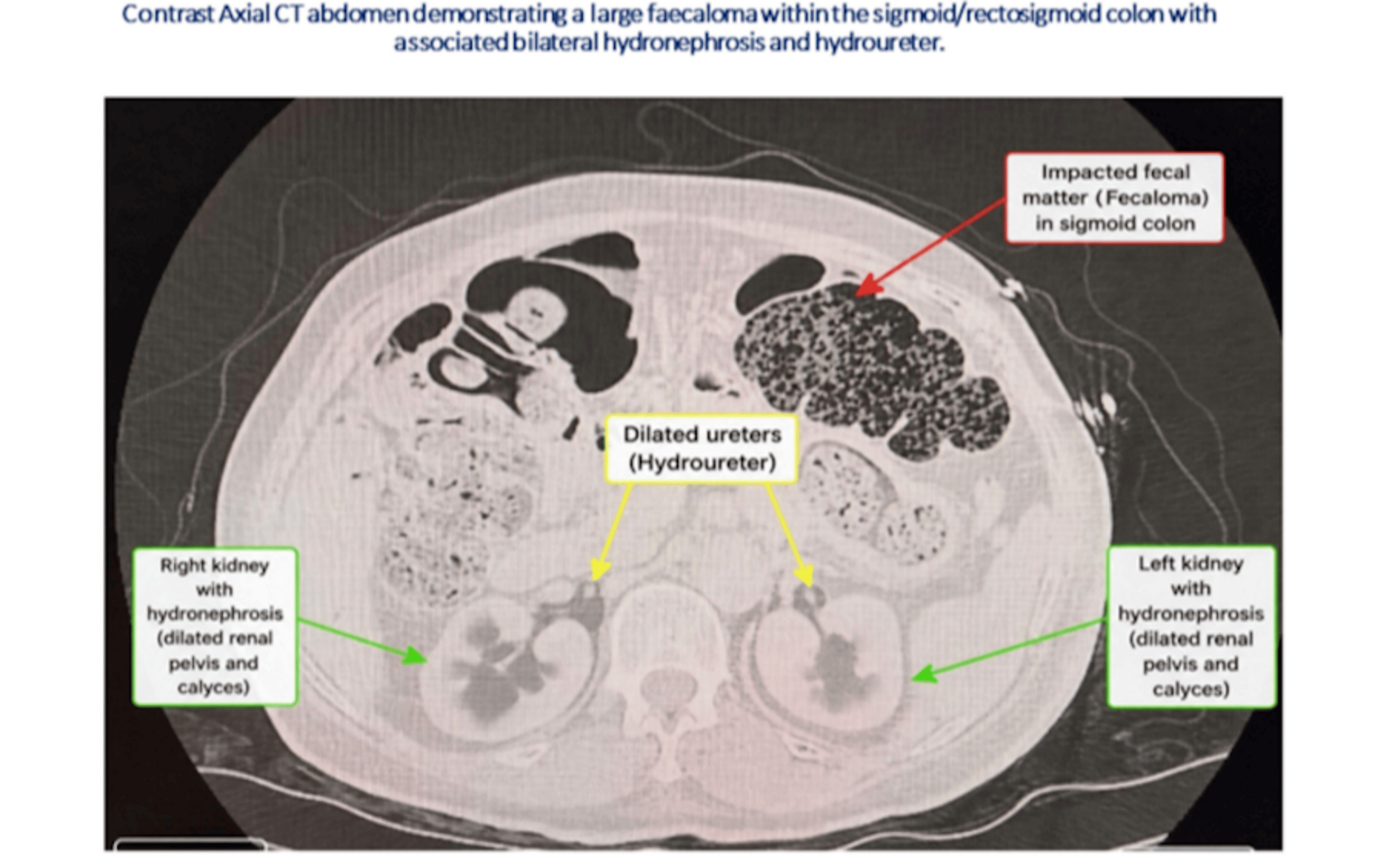
Contrast-enhanced CT of the abdomen demonstrating fecaloma with bilateral hydronephrosis and hydroureter. Axial contrast-enhanced CT of the abdomen demonstrating a fecaloma (red arrow) within the sigmoid colon, consistent with fecal impaction. There is associated bilateral hydronephrosis (green arrows). The ureters appear prominent (yellow arrow), in keeping with hydroureter measuring approximately 0.9 cm on the right and 0.8 cm on the left, in the context of bilateral hydronephrosis. Overall findings are consistent with stercoral colitis causing extrinsic compression of the distal ureters and resultant obstructive uropathy.

Because abdominal pain and urinary retention persisted, a CT scan of the abdomen and pelvis was performed. The study demonstrated no renal or ureteric calculi, and the liver, spleen, pancreas, and biliary tree appeared unremarkable, with no biliary dilatation or pneumoperitoneum to suggest perforation. In contrast, there was marked fecal loading with a large fecaloma (7.1 × 6.2 cm) within the sigmoid colon and rectum, associated with bowel wall thickening and diffuse pericolonic fat stranding, in keeping with stercoral colitis. Although diverticula were present in the sigmoid colon, the inflammatory changes were diffuse rather than focal, favoring stercoral colitis secondary to fecal impaction over acute diverticulitis. Importantly, there was pronounced extrinsic compression of the urinary bladder, with associated bilateral hydronephrosis (renal pelvic dilatation of approximately 2.0 cm on the right and 1.9 cm on the left) and hydroureter (0.9 cm on the right and 0.8 cm on the left), in the absence of intrinsic obstructing urological pathology, consistent with mass effect from the fecaloma (Figures 2, 3).

In light of these findings, the working diagnosis shifted from primary urological or alternative intra‑abdominal pathology to fecal impaction complicated by stercoral colitis causing secondary obstructive uropathy. Both surgical and urological teams were consulted. The urology team confirmed adequate urine output following catheterization and recommended conservative management without invasive intervention, while the surgical team advised initiation of a fecal disimpaction regimen.

The patient was managed conservatively with aggressive bowel decompression, including high‑dose oral osmotic laxative therapy using polyethylene glycol (macrogol 3350), commenced at four sachets daily and escalated as required, in combination with rectal phosphate enemas, resulting in prompt restoration of bowel habit. Intravenous fluids were administered to address dehydration, and empirical intravenous co‑amoxiclav was started in accordance with local guidelines in the context of rising inflammatory markers (C‑reactive protein increasing to 216 mg/L) and CT evidence of stercoral colitis concerning for evolving intra‑abdominal inflammatory or infective pathology. Following bowel decompression and ongoing conservative therapy, urinary tract obstruction resolved, and there was no recurrence of obstructive urinary symptoms after catheter removal, supporting the diagnosis of obstructive uropathy secondary to extrinsic compression from the fecaloma. The patient remains clinically stable as an inpatient, with ongoing monitoring and optimization of bowel and urinary function.

## Discussion

This case highlights an uncommon but clinically important presentation of stercoral colitis manifesting as obstructive uropathy, with bilateral hydronephrosis and hydroureter in the absence of intrinsic urological pathology. Although stercoral colitis is increasingly recognized with the wider use of CT, its extension beyond the bowel to produce clinically significant urinary tract obstruction remains infrequently reported in the literature and is therefore likely underrecognized in routine practice [1,13]. In most reported series, complications are predominantly gastrointestinal, and involvement of adjacent pelvic structures is described only in isolated case reports.

The mechanism in our patient is best understood through the natural progression of fecal impaction. Chronic stool stasis within the rectosigmoid colon leads to the formation of a fecaloma, which progressively increases intraluminal pressure. This results not only in mucosal ischemia and inflammation but, in advanced cases, in a mass effect within the relatively confined pelvic compartment. Anatomically, the close relationship between the rectosigmoid colon, urinary bladder, and distal ureters provides a plausible pathway for extrinsic compression. Unlike intrinsic obstruction, such as ureteric calculi, this represents a reversible mechanical process, where resolution of fecal loading directly relieves urinary tract obstruction, a phenomenon clearly demonstrated in our case following disimpaction [13].

Although stercoral colitis itself is uncommon, its progression to clinically significant obstructive uropathy appears to be infrequently reported rather than truly rare. Most reported complications center on perforation, ischemia, or sepsis. In contrast, urinary tract involvement has largely been confined to isolated case series and reports. Bilateral hydroureteronephrosis, as observed in our patient, suggests a significant and sustained pelvic mass effect, rather than incidental compression. Furthermore, the presenting complaint of urinary retention, rather than abdominal sepsis or peritonitis, highlights how such cases may initially be directed toward a urological diagnostic pathway, particularly in the early stages of evaluation [14].

A limited but informative body of literature supports this mechanism. Joo and Lee described acute hydronephrosis secondary to rectal fecaloma causing vesicoureteric junction compression, which resolved following bowel decompression [15]. Similarly, Knobel et al. reported bilateral hydronephrosis with acute kidney injury due to severe fecal impaction, again demonstrating reversibility with conservative measures [16]. Additional reports have described similar presentations in frail or chronically constipated patients, reinforcing the concept that fecaloma-related mass effect, although infrequently reported, is a genuine and clinically significant cause of obstructive uropathy [17]. Compared with these reports, our case is notable for the absence of prior recurrent constipation or healthcare contact, suggesting that even relatively acute fecal loading in susceptible individuals can produce significant downstream effects. This may also explain the preservation of renal function in our patient, as the obstruction was likely of short duration, in contrast to more prolonged cases where sustained back pressure can lead to significant renal impairment.

Our patient’s underlying neuropsychiatric condition and medication profile provide important context. Schizophrenia is associated with altered bowel habits, reduced symptom reporting, and delayed presentation. In addition, medications such as olanzapine and procyclidine impair gastrointestinal motility through anticholinergic and central mechanisms, predisposing to fecal stasis. While antipsychotic-associated constipation is well recognized, it is often under-monitored in routine practice, particularly in stable patients without prior gastrointestinal complaints. This case underscores how the cumulative effect of long-term therapy may remain clinically silent until an acute complication develops [18].

From a diagnostic perspective, this case also highlights the importance of CT imaging in distinguishing stercoral colitis from other causes of abdominal pathology, particularly diverticulitis. Both conditions may demonstrate bowel wall thickening and pericolonic fat stranding; however, stercoral colitis is typically characterized by diffuse involvement associated with marked fecal loading, whereas diverticulitis tends to produce more focal inflammation centered on diverticula. In our patient, the diffuse nature of inflammatory changes involving the sigmoid colon and rectum, in conjunction with a large fecaloma, favored stercoral colitis as the primary process [19,20]. Importantly, CT also allowed identification of secondary urinary tract involvement, which may not be clinically apparent.

Recognition of this mechanism has direct implications for management. In contrast to intrinsic obstructive uropathy, where urological intervention may be required, the primary therapeutic target in such cases is relief of fecal impaction. Conservative measures, including high-dose osmotic laxatives, enemas, and manual disimpaction, are often sufficient and can result in rapid resolution of both gastrointestinal and urinary manifestations. In our case, prompt initiation of a disimpaction regimen led to restoration of bowel function and improvement in urinary output, without the need for invasive urological procedures. This reinforces the importance of early multidisciplinary input and avoidance of unnecessary intervention when the underlying cause is correctly identified [21].

Finally, this case highlights a broader clinical lesson. Urinary retention and hydronephrosis are frequently approached from a urological perspective; however, extrinsic gastrointestinal causes, although uncommon, should be considered, particularly in patients with risk factors for constipation or impaired bowel motility. Failure to recognize this relationship may lead to delayed diagnosis, inappropriate intervention, or progression to more serious complications such as perforation. In this context, our case adds to the limited literature describing stercoral colitis as a reversible cause of obstructive uropathy and emphasizes the need for a more integrated, system-based diagnostic approach [22]. As a single case report, the findings are inherently limited in generalizability, and long-term follow-up was not available to assess recurrence risk. Nevertheless, given that this presentation is infrequently reported and the potential for complete reversibility with timely management, the case provides a clinically relevant contribution to existing literature.

## Conclusions

This case highlights stercoral colitis as an important, potentially reversible, and likely underrecognized cause of obstructive uropathy secondary to fecal impaction. In patients presenting with urinary retention or hydronephrosis, particularly those with neuropsychiatric comorbidities or exposure to medications that impair bowel motility, gastrointestinal pathology should remain a prominent differential consideration. Early CT imaging and prompt bowel decompression can facilitate timely diagnosis, prevent progression to severe complications, help avoid unnecessary invasive urological intervention, and support favorable clinical and functional outcomes.
